# Binary Mixtures of Some Active Pharmaceutical Ingredients with Fatty Alcohols—The Criteria of Successful Eutectic Formation and Dissolution Improvement

**DOI:** 10.3390/pharmaceutics12111098

**Published:** 2020-11-16

**Authors:** Songhee Jin, Jisun Jang, Soyeon Lee, Il Won Kim

**Affiliations:** Department of Chemical Engineering, Soongsil University, Seoul 06978, Korea; jjsong820@naver.com (S.J.); v0512@hanmail.net (J.J.); ssoyeon216@naver.com (S.L.)

**Keywords:** active pharmaceutical ingredient, fatty alcohol, eutectic, dissolution

## Abstract

Pharmaceutical eutectics are solid mixtures, where the crystals of active pharmaceutical ingredients (APIs) are finely divided in the phase-separated microstructures. The size reduction makes the eutectic formation a viable option to improve the dissolution rate of the poorly soluble APIs. In the present study, ibuprofen, naproxen, and sorafenib were investigated in terms of their phase behaviors with fatty alcohols, such as tetradecanol, octadecanol, and docosanol. Among the studied APIs, only ibuprofen was able to form eutectics with the fatty alcohols, and this was in agreement with the feasibility prediction based on the van ’t Hoff equation and solubility parameters. In vitro release behavior was significantly improved for the ibuprofen/octadecanol eutectic mixture, although the practical insolubility of octadecanol in water was the opposite of the outstanding hydrophilicity of usual eutectic formers. The feasibility prediction and the choice of eutectic formers in the present study will be useful in advancing the utility of the pharmaceutical eutectics.

## 1. Introduction

About 70–90% of drug candidates and 40% of the marketed drugs are estimated to suffer the problem of inadequate bioavailability caused by their poor aqueous solubilities [1,2]. These active pharmaceutical ingredients (APIs) are categorized as classes 2 and 4 in the biopharmaceutical classification system (BCS), and diverse approaches have been devised to solve their solubility problems [3,4,5,6]. First, intrinsic improvement of the solubility is pursued through the formation and selection of salts, cocrystals, metastable polymorphs, and amorphous phases of the APIs [3,4,5,7]. The salt formation and cocrystallization can be complementary to each other since the former requires ionizable groups and the latter usually relies on strong hydrogen bonding [5,7]. Metastable polymorphs and amorphous phases are relatively unstable high energy states that require additional stabilization strategies [4,5,6]. Second, kinetic enhancement of the dissolution rate is the goal of the size diminution where the surface area in contact with the physiological aqueous environments is enlarged [4,6]. Also, when the particle size is about below 200 nm, an additional solubility increase becomes notable [8,9]. Top-down approaches for the size reduction include milling and high-pressure homogenization, and bottom-up methods precipitation, freeze drying, and phase separation [4,6,10,11,12,13]. During the process, avoiding the aggregation of small particles is critical, which can arise from the high energy characteristics of their surfaces [6,10].

The reduction of crystal size through phase separation is of special interest because the process is thermodynamically driven, and the phase-separated drug crystals are encased and stabilized by the carrier excipients [13,14,15]. Especially, when the API and the carrier form a eutectic mixture at a specific composition, a lamellar microstructure is generated the API and carrier nanocrystals sandwiching one another [13,15,16]. The resulting enhancement of the drug dissolution has been long recognized in pharmaceutics. Diverse systems have been studied, and the classic examples include sulfathiazole/urea, griseofulvin/succinic acid, and fenofibrate/poly(ethylene glycol) [13,15,17,18,19,20]. While the effectiveness of the eutectics is evident with the many successful examples, there are some issues to address to fully utilize the process of eutectic formation. First, the characteristics of the effectual eutectic formers (the eutectic-inducing carrier materials) have been investigated to predict the successful pairs of API/eutectic former. The studies have been approached from both thermodynamic and molecular viewpoints, and the theories need to be substantiated with more diverse cases [13,15,21,22]. Second, the hydrophilicity of the eutectic formers and their aqueous solubilization have been considered as contributing features in the previous studies [4,13,14]. Still, it is not obvious if it is a necessary condition for the dissolution improvement of APIs. In addition, the biocompatibility of the eutectic formers is another factor to consider carefully [23].

**Figure 1:**
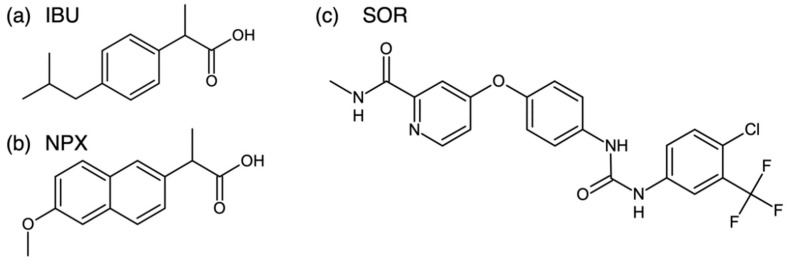
Molecular structures of (**a**) ibuprofen (IBU), (**b**) naproxen (NPX), and (**c**) sorafenib (SOR).

In the present study, we explored the eutectic formation of APIs with saturated fatty alcohols of low toxicity [24]. Specifically, tetradecanol (TD, CH_3_(CH_2_)_13_OH), octadecanol (OD, CH_3_(CH_2_)_17_OH), and docosanol (DC, CH_3_(CH_2_)_21_OH) were used. TD (myristyl alcohol) and OD (stearyl alcohol) are common pharmaceutical excipients used in various formulations including oral [25,26]. They are also permitted food additives [27]. DC (behenyl alcohol) is used for topical treatment with its antiviral activity [28]. Also, TD, OD, and DC are practically insoluble in water [24]. The model APIs in this study are ibuprofen (IBU), naproxen (NPX), and sorafenib (SOR), which are well-known for their limited solubilities (Figure 1) [11,21,22]. In summary, the combinations of the fatty alcohols and APIs were studied to understand the conditions to successfully form the eutectic structures. When successful, we aimed for the optimal eutectic pairs for the improvement of the API release behavior.

## 2. Materials and Methods

### 2.1. Materials

Three active pharmaceutical ingredients (APIs) were ibuprofen (IBU: 99%, Alfa Aesar, Tewksbury, MA, USA), naproxen (NPX: ≥ 98%, Tokyo Chemical Industry, Tokyo, Japan), and sorafenib (SOR: ≥ 99%, Jinan Rouse Industry, Jinan, China). All fatty alcohols were purchased from Sigma-Aldrich (St. Louis, MO, USA): 1-tetradecanol (TD, CH_3_(CH_2_)_13_OH: ≥ 95%), 1-octadecanol (OD, CH_3_(CH_2_)_17_OH: 99%), and 1-docosanol (DC, CH_3_(CH_2_)_21_OH: 98%).

To make a fed state simulated intestinal fluid (FeSSIF), FaSSIF/FeSSIF/FaSSGF powder was purchased from Biorelevant (London, UK). Sodium chloride (NaCl, ≥ 99.5%), sodium hydroxide (NaOH, ≥ 97%), and acetic acid (≥ 99%) were from Sigma-Aldrich. Further, 1 M HCl (aq) and 1 M NaOH (aq) were obtained from Samchun Chemical (Seoul, Korea) and Daejung Chemical (Gyeonggi, Korea), respectively. Deionized water (DI water, resistivity > 18.2 MΩ·cm) was from a Direct-Q3 water purification system (Millipore, Burlington, MA, USA).

### 2.2. Thermal Analysis and Melt Crystallization

Phase behaviors of the mixtures of APIs (IBU, NPX, and SOR) and fatty alcohols (TD, OD, and DC) were investigated using a differential scanning calorimeter (DSC: DSC3 STARe system, Mettler-Toledo, Columbus, OH, USA). DSC was calibrated before experiments for temperature and enthalpy using indium and zinc. Sample mixtures were lightly ground with an agate mortar and pestle, and 2–3 mg of the powders were used for each DSC run (40 μL Al pan with a pinhole-punched lid under nitrogen gas atmosphere, heating rate 10 °C/min). Each sample measurement was repeated in triplicate.

Melt crystallized samples for further characterization were prepared as follows. Each API and fatty alcohol were separately ground for 2 min with an agate mortar and pestle, which was used to prepare the API/fatty alcohol mixtures in the desired ratios. The mixtures were melted by heating on Al foil using a hot plate (HS-180, Misung Scientific, Gyeonggi, Korea). The completely melted mixtures were moved to a refrigerator (4 °C) and crystallized. After 24 h, the crystalline samples were lightly ground for 2 min and used for further analyses.

Recrystallization behaviors of some melt crystallized mixtures were observed using an optical microscope (OM: BX-51, Olympus, Tokyo, Japan) coupled with a hot stage (FP90, Mettler-Toledo, Columbus, OH, USA). Typically, a melt crystallized sample between a glass slide and a cover glass was heated to remelt completely. Then, the recrystallization process was monitored during cooling under cross polarization equipped with the first-order retardation plate.

### 2.3. Characterization of Melt Crystallized API/Fatty Alcohol Mixtures

In vitro release behavior was studied in FeSSIF (pH 5.0, 37 °C) using a USP type II apparatus (paddle) at 100 rpm (RC-3 dissolution tester, Minhua Pharmaceutical Machinery, Shanghai, China). FeSSIF was prepared following the recipe provided by the supplier (Biorelevant, London, UK). It contained sodium taurocholate (15 mM), lecithin (3.75 mM), sodium chloride (203 mM), sodium hydroxide (101 mM), and acetic acid (144 mM). For each dissolution test, an appropriate amount of powder mixture was added to a 500 mL FeSSIF solution to have IBU 2 mg/mL. The 3 mL aliquots of the solution were withdrawn after 5, 10, 20, 30, 40, 60, 90, and 120 min, and the equal amount of the fresh FeSSIF solution was immediately added to maintain the total volume constant. The UV absorbance of each aliquot was measured at 264 nm (V730, Jasco, Tokyo, Japan) after filtering through a 0.20 μm PTFE filter (Advantec, Tokyo, Japan), and it was converted to IBU concentration using a pre-constructed calibration curve. All release experiments were independently repeated in triplicate.

X-ray diffraction (XRD) was performed on the melt crystallized samples using a D2 PHASER diffractometer (Bruker AXS, Billerica, MA, USA). A 2*θ* range of 5 to 35° was scanned at 0.02° increment (scanning rate 1°/min) with CuKα radiation (λ = 1.5406 Å) at 30 kV and 10 mA. The operation was in the *θ*-*θ* mode, and a zero-background holder (Bruker AXS) was used for better sensitivity.

## 3. Results and Discussion

### 3.1. Melting Behaviors of API/Fatty Alcohol Mixtures

**Figure 2:**
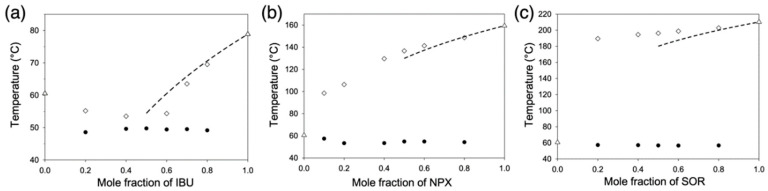
Melting diagrams of (**a**) IBU/octadecanol (OD), (**b**) NPX/OD, and (**c**) SOR/OD mixtures. Dotted lines indicate ideal behavior calculated with the van’t Hoff equation; empty triangles melting points of pure components; empty diamonds liquidus temperatures; filled circles solidus temperatures.

Nine melting diagrams of the combinatorial mixtures of the three APIs and the three fatty alcohols were constructed based on DSC thermograms (Figure 2, Figures S1–S6). Figure 2 shows the representative diagrams of APIs with OD, and those with TD and DC are qualitatively similar within the same APIs (Figures S1–S3). IBU formed eutectic mixtures with all three fatty alcohols (Figure 2a and Figure S1). The approximate eutectic compositions were IBU/TD = 3:7, IBU/OD = 5:5, and IBU/DC = 6:4, and their eutectic temperatures were ca. 30, 49, and 59 °C, respectively. In contrast, NPX and SOR formed no eutectic mixture with any fatty alcohol (Figure 2b,c and Figures S2 and S3).

Analysis of the melting diagrams based on the van ’t Hoff equation revealed high compatibility between IBU and the fatty alcohols in the liquid phases:

(1) $$\frac{1}{T_{fus}}=\frac{1}{T_{fus,API*}}-\frac{R}{\Delta H_{fus,API*}}\ln\left(\gamma_{API}x_{API}\right),$$

where *x_API_*, *γ_API_*, and *R* are the mole fraction of an API, activity coefficient, and gas constant, respectively; ΔH_fus,API*_ and T_fus,API*_ are the molar enthalpy of fusion and melting point of the pure API; *T*_fus_ is the melting point at *x_API_* [29]. The dotted lines in the melting diagrams in Figure 2 and Figures S1–S3 represent ideal behavior in the API-rich regions, where the activity coefficient of API in the liquid solution (*γ_API_*) is unity. Only IBU/fatty alcohol cases showed negative deviations from the ideal behavior indicating favorable interactions between the API and the fatty alcohols [30]. For example, the *γ_API_* value at *x_API_* = 0.8 was 0.97 with OD, and it became smaller with more OD (0.94 and 0.83 at *x_API_* = 0.7 and 0.6, respectively) showing larger deviations from the ideal behavior. In contrast, NPX and SOR did not show similar negative deviations with any fatty alcohols.

An alternative approach with the van ’t Hoff equation has been provided in the form of an index *I*_c_ [21]. A slightly modified equation for the *I*_c_ can be written as follows if the assumption of marginal melting point depression in the original equation is lifted [29]:

(2) $$I_{c}=\frac{\Delta H_{fus,API*}}{R}\left(\frac{1}{T_{fus,fa}}-\frac{1}{T_{fus,API*}}\right),$$

where *T_fus,fa_* is the melting point of pure fatty acid, and the others are as previously defined for Equation (1). *I*_c_ for IBU was ca 1.15, 0.51, and 0.13 with TD, OD, and DC, respectively. *I*_c_ for NPX was 3.62, 2.81, and 2.34 with TD, OD, and DC, respectively. *I*_c_ for SOR was 5.66, 4.68, and 4.10 with TD, OD, and DC, respectively. (Note that the experimental data are in Supplementary Table S1). Since the eutectic formation has been predicted when *I*_c_ < 2.5, the probability of eutectic formation was estimated high for IBU, marginal for NPX, and limited for SOR [21].

Compatibility between APIs and fatty alcohols was also estimated by translating their molecular structures into the solubility parameters using a group contribution method [31]. Solubility parameters have been used to predict the miscibility of the various compounds, such as solvents, polymers, and APIs [32,33]. The values of the solubility parameters (δ) for the fatty alcohols in this study are TD 18.87, OD 18.61, and DC 18.44 MPa^1/2^. Those for the APIs are IBU 20.95, NPX 23.41, and SOR 29.22 MPa^1/2^. The proximity between the δ values of fatty alcohols and IBU (Δδ ⪅ 2.5 MPa^1/2^) is qualitatively consistent with the experimental results of the melting diagrams and their analysis. The differences of solubility parameters (Δδ) were over about 4.5 MPa^1/2^ between NPX and fatty alcohols, and 10.3 MPa^1/2^ between SOR and fatty alcohols.

More quantitative analysis could be performed using a simple and useful criterion developed for the prediction of miscibility at temperature *T* (K) using solubility parameters (*δ_solute_* and *δ_solvent_* in MPa^1/2^) and molar volume (*V_solute_* in cm^3^/mol) [34]:

(3) $$4.80\sqrt{T}\geq \sqrt{V_{solute}}\;\left|\delta_{solvent}-\delta_{solute}\right|.$$

If *T* = 300 (and 350) K is taken for simplicity since the melting temperatures of the fatty alcohols are 313, 334, and 347 K for TD, OD, and DC, respectively, the *δ_solvent_* (*δ_fatty alcohol_*) criteria for the APIs (*V_solute_* = 200.3, 192.3, and 319.5 cm^3^/mol for IBU, NPX, and SOR, respectively) in the present study become as follows [35]: 15.08 (14.60) ≤ *δ_fatty alcohol_* (MPa^1/2^) ≤ 26.82 (27.30) for IBU; 17.41 (16.93) ≤ *δ_fatty alcohol_* (MPa^1/2^) ≤ 29.41 (29.89) for NPX; 24.57 (24.20) ≤ *δ_fatty alcohol_* (MPa^1/2^) ≤ 33.87 (34.24) for SOR. The results predict the compatibility of the APIs with the current fatty alcohols (*δ_fatty alcohol_* ~ 18 MPa^1/2^) in the order IBU (high) > NPX (borderline) >> SOR (no). The advantage of the solubility parameter approach is no use of experimental data from the melting point diagram construction. The general utility of this approach is currently under investigation with structurally more diverse excipients. 

Overall, the analysis of the melting behaviors of the nine API-fatty alcohol mixtures revealed that the alcohol-type excipients (TD, OD, and DC) exhibited eutectic behavior only with IBU among the APIs in the present study. The high compatibility of IBU was confirmed by the analyses of both van ’t Hoff equation (*γ* < 1) and solubility parameters (Δ*δ* ⪅ 2.5 MPa^1/2^). From now on, further structure-property characterization will be limited to the eutectic-forming IBU cases.

### 3.2. Crystallization Behaviors of IBU/Fatty Alcohol Mixtures

**Figure 3:**
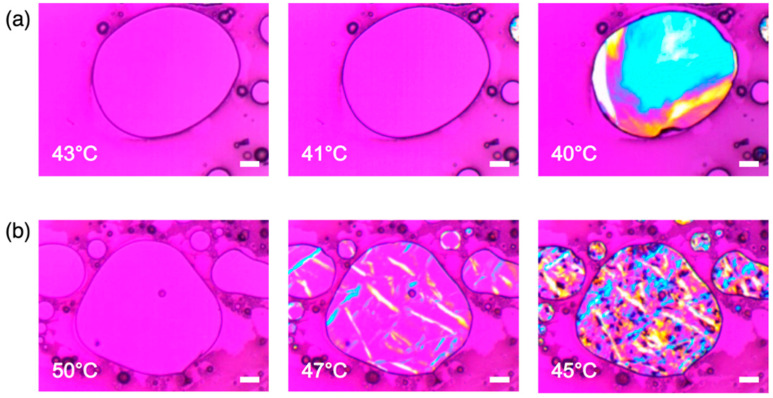
OM micrographs of IBU/OD mixtures at the compositions of IBU/OD (**a**) 5:5 (eutectic mixture) and (**b**) 2:8. Cooling crystallization behaviors were shown under cross polarization. All scale bars are 100 μm.

The crystallization behaviors of IBU/fatty alcohol mixtures were studied after melting at 90 °C (< 1 min) using OM coupled with a hot stage under cross polarization. Representative compositions for IBU/OD and IBU/DC are shown in Figure 3 and Supplementary Figure S7, respectively. IBU/TD case was omitted because the solid structure was thermally unstable its eutectic temperature 30 °C being too close to room temperature.

Figure 3 shows the clear differences between the IBU/OD crystallization behaviors at the eutectic and non-eutectic compositions. Figure 3a (IBU/OD = 5:5 eutectic mixture) shows the abrupt appearance of the birefringence at 40–41 °C in the entire domain demonstrating sweeping crystallization with the supercooling about 8–9 °C (eutectic temperature 49 °C). In contrast, Figure 3b (IBU/OD = 2:8) displays the initial nucleation of OD crystals (47 °C) followed by its growth and eutectic crystallization (45 °C). As a result, the eutectic mixture displays subtle fibrillar features within relatively homogeneous domains, and the non-eutectic mixture shows easily discernable microstructures. (The gross color difference in the birefringence at the eutectic composition is probably in part due to the local thickness variations of the sample sandwiched between the glass slide and the cover glass [36].) The crystallization behaviors are in accordance with the experimentally determined melting diagram of IBU/OD and the fact that a lamellar structure develops during the solidification at the eutectic composition to minimize the distance of molecular diffusion during the phase separation [13,15,16]. Note that in the IBU/OD = 2:8 example, 3/4 OD should crystallize as separate domains and 1/4 OD forms a eutectic structure with the same amount (mole) of IBU since the eutectic composition is IBU/OD = 1:1.

Similarly, Supplementary Figure S7a (IBU/DC = 6:4 eutectic mixture) shows the sudden and extensive crystallization at 52–54 °C with the supercooling about 5–7 °C (eutectic temperature 59 °C). Also, Supplementary Figure S7b (IBU/DC = 2:8) shows the initial DC nucleation (65 and 62 °C) followed by the crystal growth (60 °C). Here, 5/6 DC should crystallize as separate domains and 1/6 DC forms a eutectic structure with a 50% larger amount (mole) of IBU since the eutectic composition is IBU/DC = 3:2.

Overall, the observed crystallization behavior conforms to the experimentally determined melting diagrams and the classic behavior of binary eutectic mixtures. The implications of the phase-separated structures at the eutectic compositions were evaluated in terms of the IBU release behavior as described in the following section.

### 3.3. In Vitro Release Behaviors of IBU/Fatty Alcohol Mixtures

**Figure 4:**
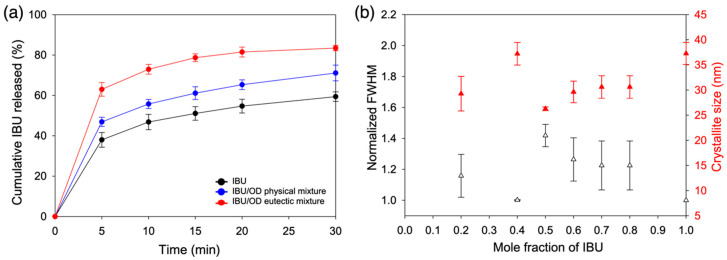
(**a**) Dissolution profiles (FeSSIF, *n* = 3) of the IBU/OD eutectic mixture (5:5) in comparison of physical mixture and neat IBU. (**b**) Microstructure analysis using peak broadening of X-ray diffraction peaks. Crystallite size was calculated using (2 0 0), (2 1 0), and (2 1 −1) diffraction peaks with the use of Scherrer equation, and the full width at half maximum (FWHM) was shown as normalized against the narrowest peaks (neat IBU).

The IBU release behaviors from the IBU/OD and IBU/DC eutectic mixtures are shown in Figure 4 and Supplementary Figure S8, respectively. Again, IBU/TD was omitted due to its thermal instability caused by its eutectic point (30 °C) close to room temperature. Dissolution media was FeSSIF, and three different samples were tested for each eutectic mixture. The eutectic mixtures were the melt crystallized samples after mixing IBU and fatty alcohols at the eutectic compositions. The physical mixtures were simple combinations of individually melt crystallized components (IBU and fatty alcohols) at the eutectic compositions. Neat IBU with the same thermal history served as a negative control.

The highest dissolution rate was observed from the IBU/OD eutectic mixtures (Figure 4a). Especially, the initial dissolution rate (< 5 min) was dramatically enhanced. After 5 min, the released portion of IBU from the IBU/OD eutectic mixture was about 63%, compared to 38% for the negative control (IBU only) and 47% for the simple physical mixture at the eutectic composition. After 30 min, ca. 83% IBU was released from the eutectic mixture, whereas about 59% and 71% were released from the negative control and the physical mixture, respectively. 

The dramatic increase of the initial release rate was attributed to the micro- and nano-structures intrinsic to the melt crystallized eutectics. The OM results (Figure 3a) without showing easily discernable microcrystalline domains suggested the reduced IBU domain size due to the lamellar structure formation. Further nanostructure analysis was performed via XRD assisted by the Scherrer equation [11,37]:

(4) $$L=\frac{K\lambda }{\beta \;cos\theta }\;$$

where crystallite size *L* is calculated as a function of the full width at half maximum (FWHM, *β*) of the diffraction peak at angle 2*θ* with the X-ray wavelength *λ* (1.5406 Å) and a constant *K* (0.9). Νote that the crystallite size obtained from the XRD analysis is not a particle size, but rather the average size of the single crystalline sections within a larger entity [11,37]. 

Figure 4b shows the crystallite size of the melt crystallized IBU/OD mixtures at various compositions. The analysis was based on the {2 0 0}, {2 1 0}, and {2 1 −1} diffraction peaks of IBU, which did not overlap with the diffraction peaks of OD (Supplementary Figure S9a) [38]. The crystallite size was the smallest (average 26.3 nm) for the melt crystallized eutectic mixture (IBU/OD = 5:5). That for the neat IBU with the same thermal history was ca. 42% bigger (average 37.3 nm), which corresponds to the 29% decrease of the surface area if spherical shapes are assumed for simplicity. All the other IBU/OD compositions displayed a larger IBU crystallite size than the eutectic mixture. The relatively small crystallite size at the lowest IBU content (IBU/OD = 2:8) was probably due to the fact that the crystallization space was already populated with OD crystals making the subsequent IBU crystal growth within the minor eutectic phase hindered. Note that the FWHM was shown as normalized against the narrowest peaks (neat IBU). 

**Figure 5:**
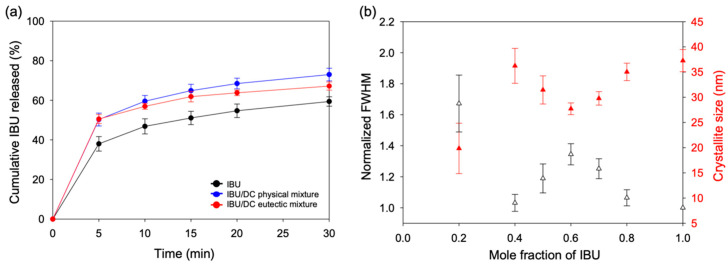
(**a**) Dissolution profiles (FeSSIF, *n* = 3) of the IBU/DC eutectic mixture (6:4) in comparison of physical mixture and neat IBU. (**b**) Microstructure analysis using peak broadening of X-ray diffraction peaks. Crystallite size was calculated using (2 0 0), (2 1 0), and (2 1 −1) diffraction peaks with the use of Scherrer equation, and the FWHM was shown as normalized against the narrowest peaks (neat IBU).

Analogous release results for IBU/DC are shown in Figure 5a. The initial dissolution rate was enhanced to release 51% IBU at 5 min for the eutectic mixture (IBU/DC = 6:4) compared to 38% for the negative control (IBU only). The physical mixture was comparable to the eutectic mixture with a 50% IBU release after 5 min. The release after 30 min was also comparable between the eutectic mixture and the physical mixture as 67% and 73%, respectively. 

Figure 5b shows the crystallite size of the melt crystallized IBU/DC mixtures. The analysis was again based on the {2 0 0}, {2 1 0}, and {2 1 −1} diffraction peaks of IBU (Supplementary Figure S9b). The crystallite size was average 27.7 nm for the melt crystallized eutectic mixture (IBU/DC = 6:4). Most other IBU/DC compositions displayed a larger IBU crystallite size. The small crystallite size at the lowest IBU content (IBU/DC = 2:8) was again (as was in IBU/OD) probably because of the hindered IBU crystal growth. The FWHM values were shown as normalized against the narrowest peaks (neat IBU).

Overall, the most successful enhancement of IBU release was achieved through the IBU/OD eutectic mixture. While TD and DC also showed the behavior of binary eutectic mixtures with IBU, these eutectic formers proved not as ideal as OD. IBU/TD suffered from thermal instability because the initially low melting point of TD (40 °C) contributed to the eutectic point (30 °C) near room temperature. The release enhancement of IBU/DC was not as much as IBU/OD probably because DC with higher hydrophobicity (DC, CH_3_(CH_2_)_21_OH vs. OD, CH_3_(CH_2_)_17_OH) counteracted the IBU release from the lamellar structures by partially functioning as a barrier.

## 4. Conclusions

In summary, some API/fatty alcohol mixtures were studied to identify successful pairs for eutectic formation. IBU, NPX, and SOR of limited aqueous solubility were chosen as model APIs, and TD, OD, and DC were the fatty alcohol excipients. Only IBU formed eutectic mixtures with the fatty alcohols, and this could be explained by its high compatibility. Also, the high compatibility of IBU with fatty alcohols could be predicted by the approaches based on van ’t Hoff equation and solubility parameters. Among the successful eutectic mixtures, IBU/OD proved optimal for the enhancement of IBU release (over 60% increase compared to neat IBU based on 5 min dissolution). The improvement is intriguing especially because the fatty alcohols are practically insoluble in water. The conventional eutectic formers for the dissolution enhancement have been mostly hydrophilic. We believe the current study helps to expand the possible choices of eutectic formers when multiple properties need to be optimized [13,39]. More diverse cases of pharmaceutically acceptable excipients are currently under investigation to further advance the utility of the eutectic formation.

## Supplementary Materials

The following are available online at https://www.mdpi.com/1999-4923/12/11/1098/s1, Table S1: Molecular weight, melting temperatures, and enthalpy of fusion for active pharmaceutical ingredients and fatty alcohols, Figure S1: Melting diagrams of (a) IBU/TD, (b) IBU/OD, and (c) IBU/DC mixtures, Figure S2: Melting diagrams of (a) NPX/TD, (b) NPX/OD, and (c) NPX/DC mixtures, Figure S3: Melting diagrams of (a) SOR/TD, (b) SOR/OD, and (c) SOR/DC mixtures, Figure S4: DSC thermograms of (a) IBU/TD, (b) IBU/OD, and (c) IBU/DC mixtures, Figure S5: DSC thermograms of (a) NPX/TD, (b) NPX/OD, and (c) NPX/DC mixtures, Figure S6: DSC thermograms of (a) SOR/TD, (b) SOR/OD, and (c) SOR/DC mixtures, Figure S7: OM micrographs of IBU/DC mixtures at the compositions of IBU/DC (a) 6:4 (eutectic mixture) and (b) 2:8, Figure S8: DSC thermograms of (a) IBU/OD and (b) IBU/DC during the cooling from 90 °C, Figure S9: XRD patterns of (a) IBU/OD and (b) IBU/DC mixtures, Figure S10: Dissolution profiles (FeSSIF, *n* = 3) of (a) IBU/OD eutectic mixture (5:5) and (b) IBU/DC eutectic mixture (6:4) in comparison of physical mixtures and pure IBU.
