# Supplementary material for: Binary Mixtures of Some Active Pharmaceutical Ingredients with Fatty Alcohols—The Criteria of Successful Eutectic Formation and Dissolution Improvement
Source: Pharmaceutics. 2020 Nov 16;12(11):1098. doi: 10.3390/pharmaceutics12111098 (PMC7698041; doi:10.3390/pharmaceutics12111098)
Supplement: Supplementary file 1 [file pharmaceutics-12-01098-s001.pdf]

pharmaceutics

Binary Mixtures of Some Active Pharmaceutical Ingredients with Fatty Alcohols—The Criteria of Successful Eutectic Formation and Dissolution Improvement

Songhee Jin, Jisun Jang, Soyeon Lee and Il Won Kim *

Table S1. Molecular weight, melting temperatures, and enthalpy of fusion for active pharmaceutical ingredients (APIs) and fatty alcohols in the present study.

		MW (g/mol)	T_{fus} (K)	ΔH_{fus} (J/g)	ΔH_{fus} (kJ/mol)
APIs	IBU	206.29	351.98	131.90	27.21
	NPX	230.26	432.48	148.20	34.12
	SOR	464.83	483.32	90.11	41.89
Fatty alcohols	TD	214.39	313.15	220.90	47.36
	OD	270.49	333.65	251.20	67.95
	DC	326.61	346.98	260.50	85.08

pharmaceutics

MDPI

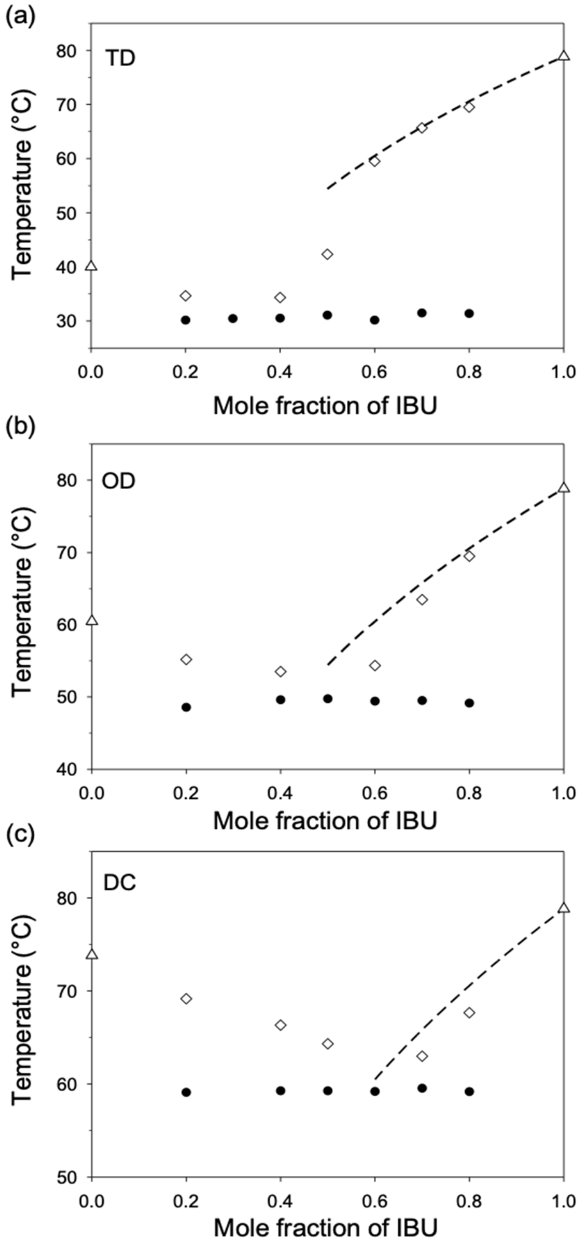

Figure S1. Melting diagrams of (a) IBU/TD, (b) IBU/OD, and (c) IBU/DC mixtures. Dotted lines indicate ideal behavior calculated with the van't Hoff equation; empty triangles melting points of pure components; empty diamond liquidus temperatures; filled circle solidus temperatures.

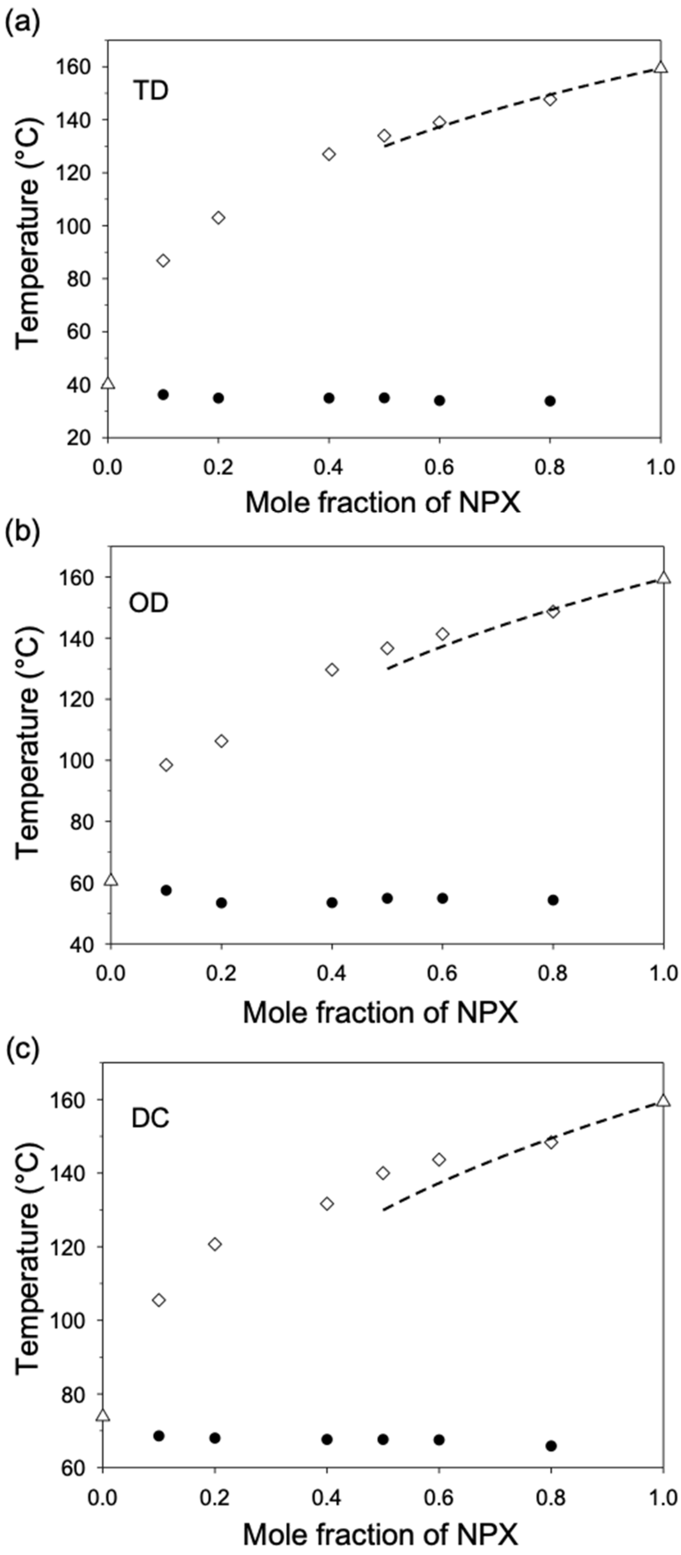

Figure S2. Melting diagrams of (a) NPX/TD, (b) NPX/OD, and (c) NPX/DC mixtures. Dotted lines indicate ideal behavior calculated with the van't Hoff equation; empty triangles melting points of pure components; empty diamond liquidus temperatures; filled circle solidus temperatures.

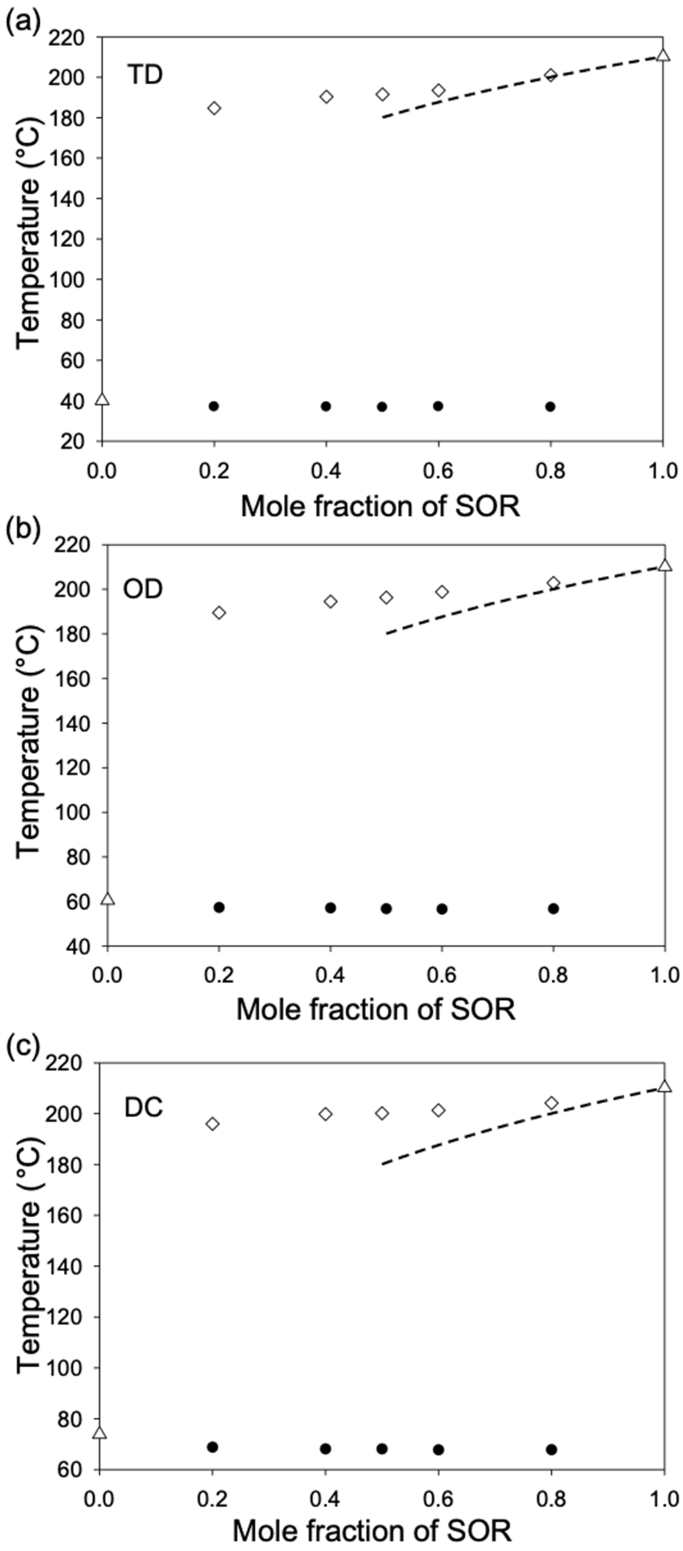

Figure S3. Melting diagrams of (a) SOR/TD, (b) SOR/OD, and (c) SOR/DC mixtures. Dotted lines indicate ideal behavior calculated with the van't Hoff equation; empty triangles melting points of pure components; empty diamond liquidus temperatures; filled circle solidus temperatures.

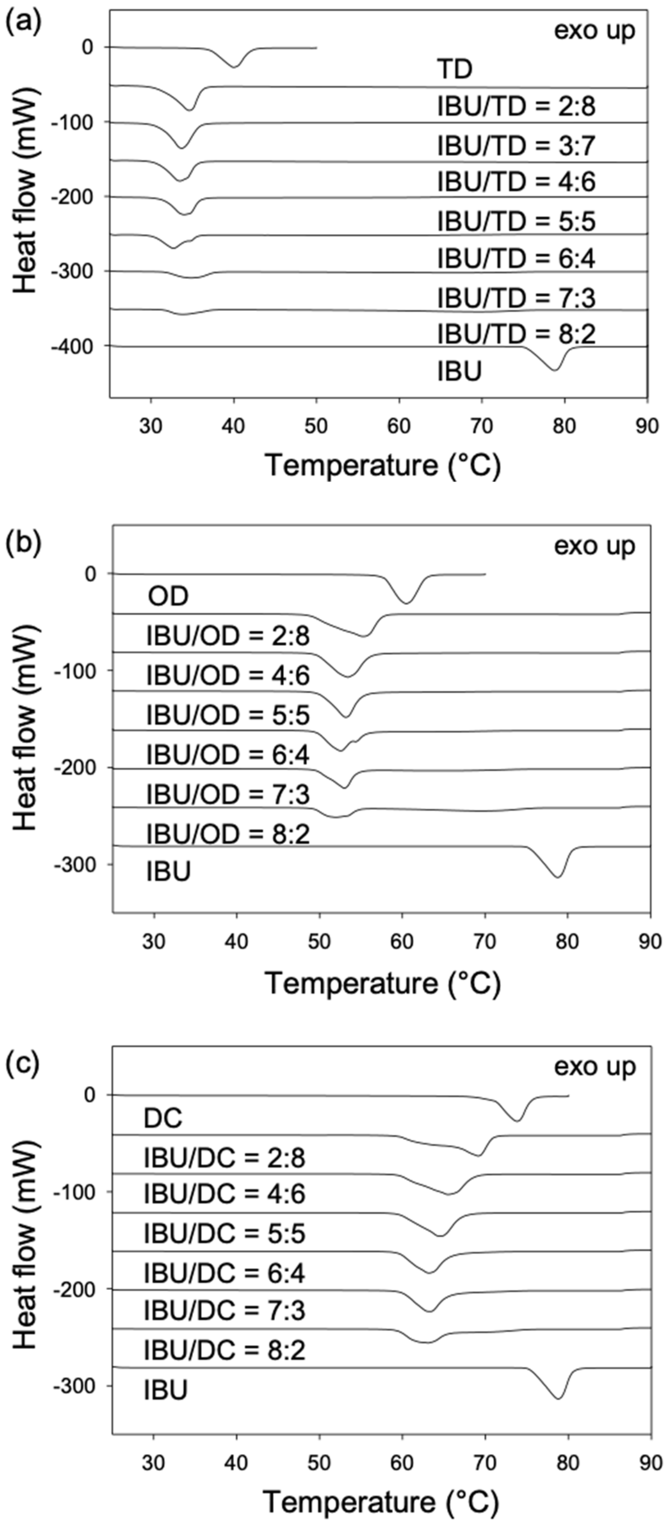

Figure S4. DSC thermograms of (a) IBU/TD, (b) IBU/OD, and (c) IBU/DC mixtures.

pharmaceutics

MDPI

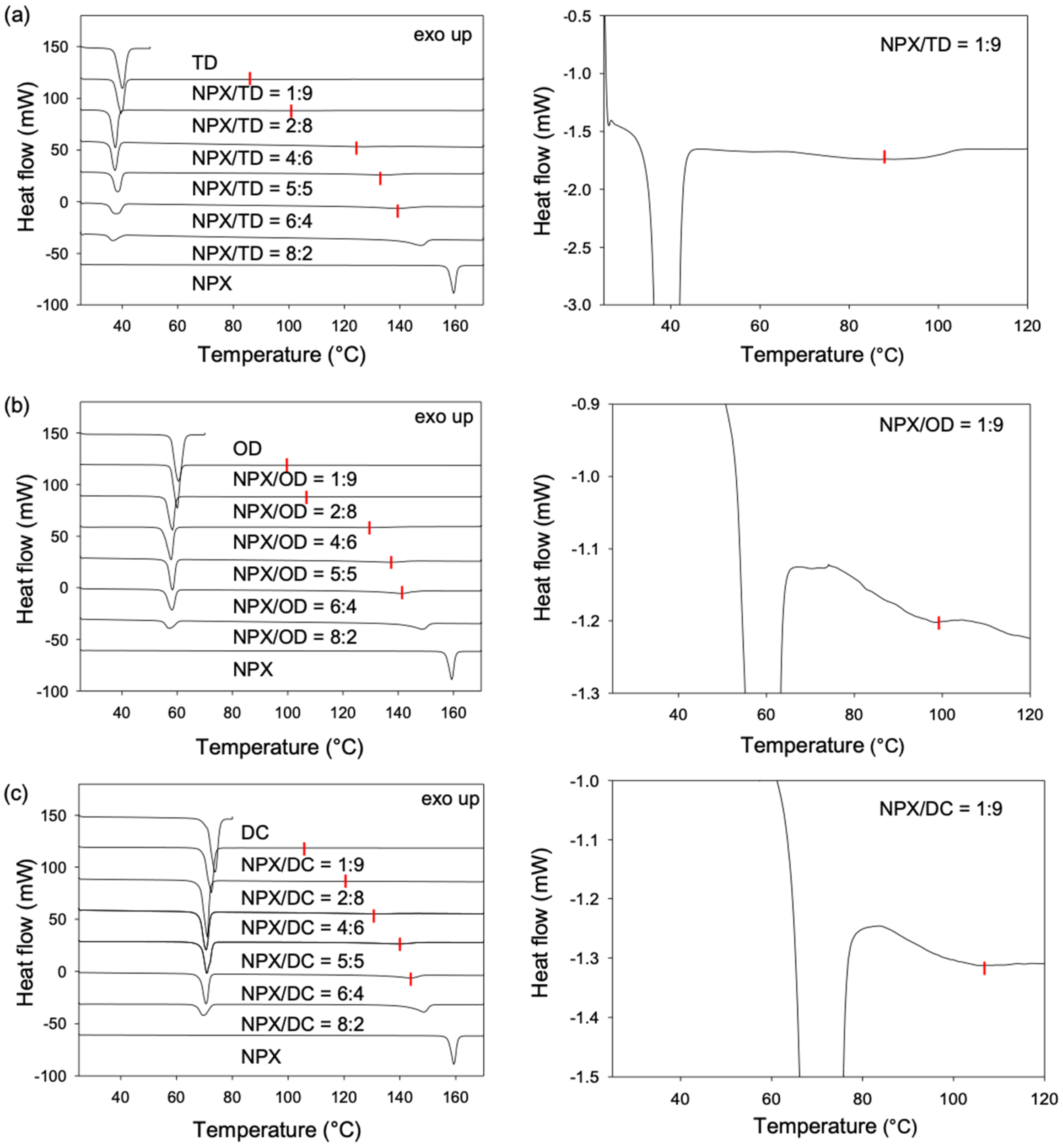

Figure S5. DSC thermograms of (a) NPX/TD, (b) NPX/OD, and (c) NPX/DC mixtures.

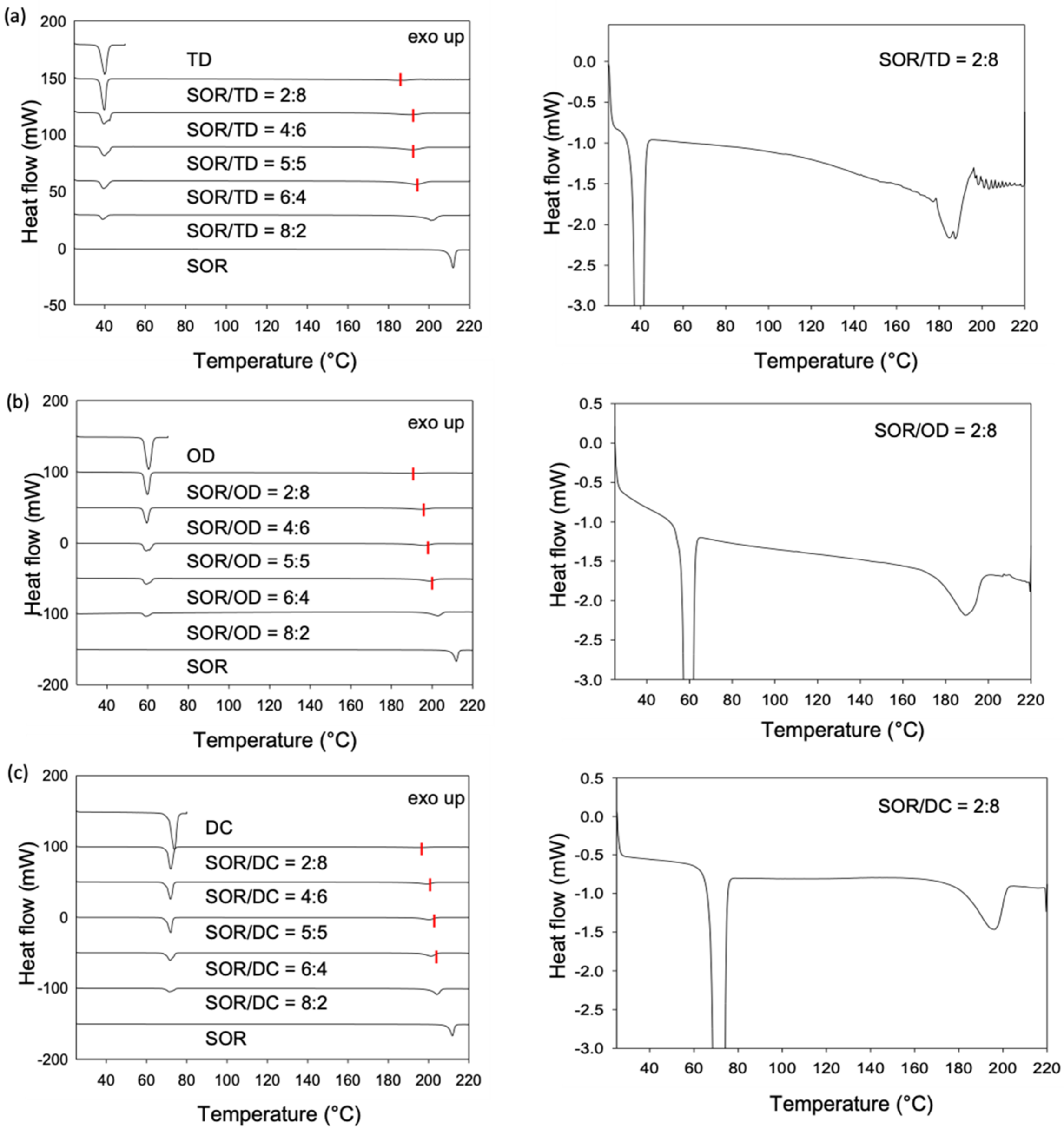

Figure S6. DSC thermograms of (a) SOR/TD, (b) SOR/OD, and (c) SOR/DC mixtures.

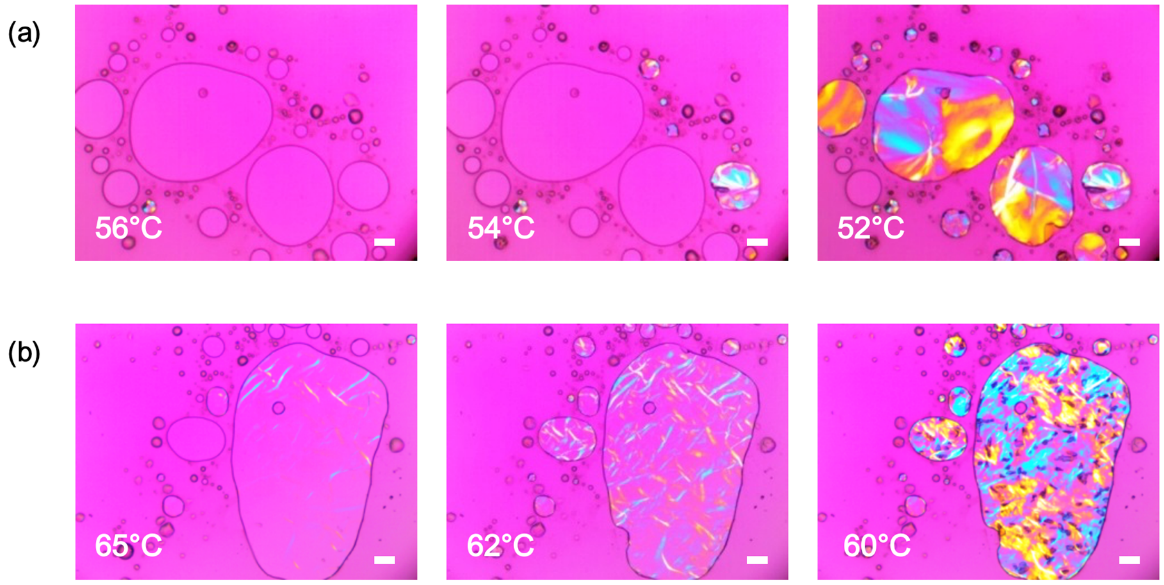

Figure S7. OM micrographs of IBU/DC mixtures at the compositions of IBU/DC (a) 6:4 (eutectic mixture) and (b) 2:8. Cooling crystallization behaviors were shown under cross polarization. All scale bars are 100 μm..

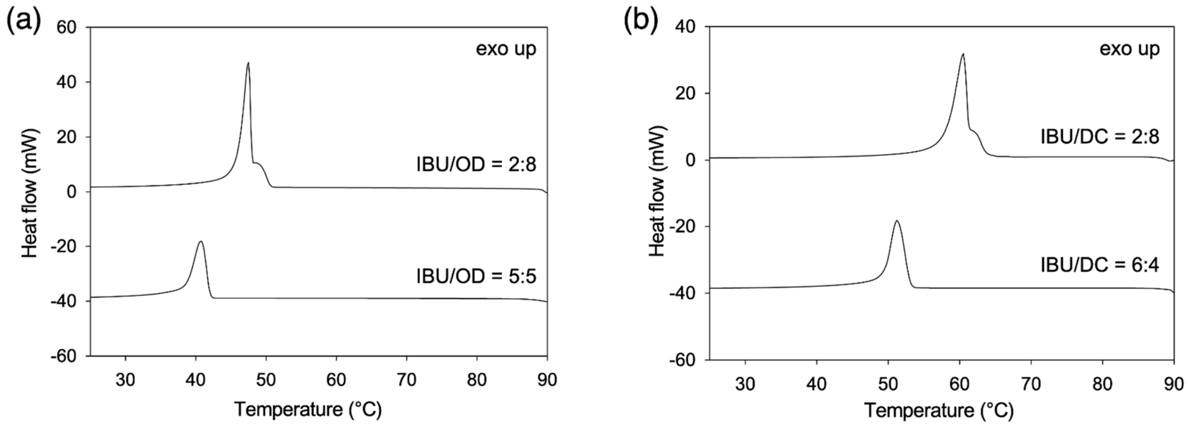

Figure S8. DSC thermograms of (a) IBU/OD and (b) IBU/DC during the cooling from 90 ºC (10 ºC/min) showing one crystallization exotherm for the eutectic mixtures (IBU/OD = 5:5 and IBU/DC = 6:4) and two exotherms for the other compositions as observed in hot-stage microscopy (see Figures 3 and S7).

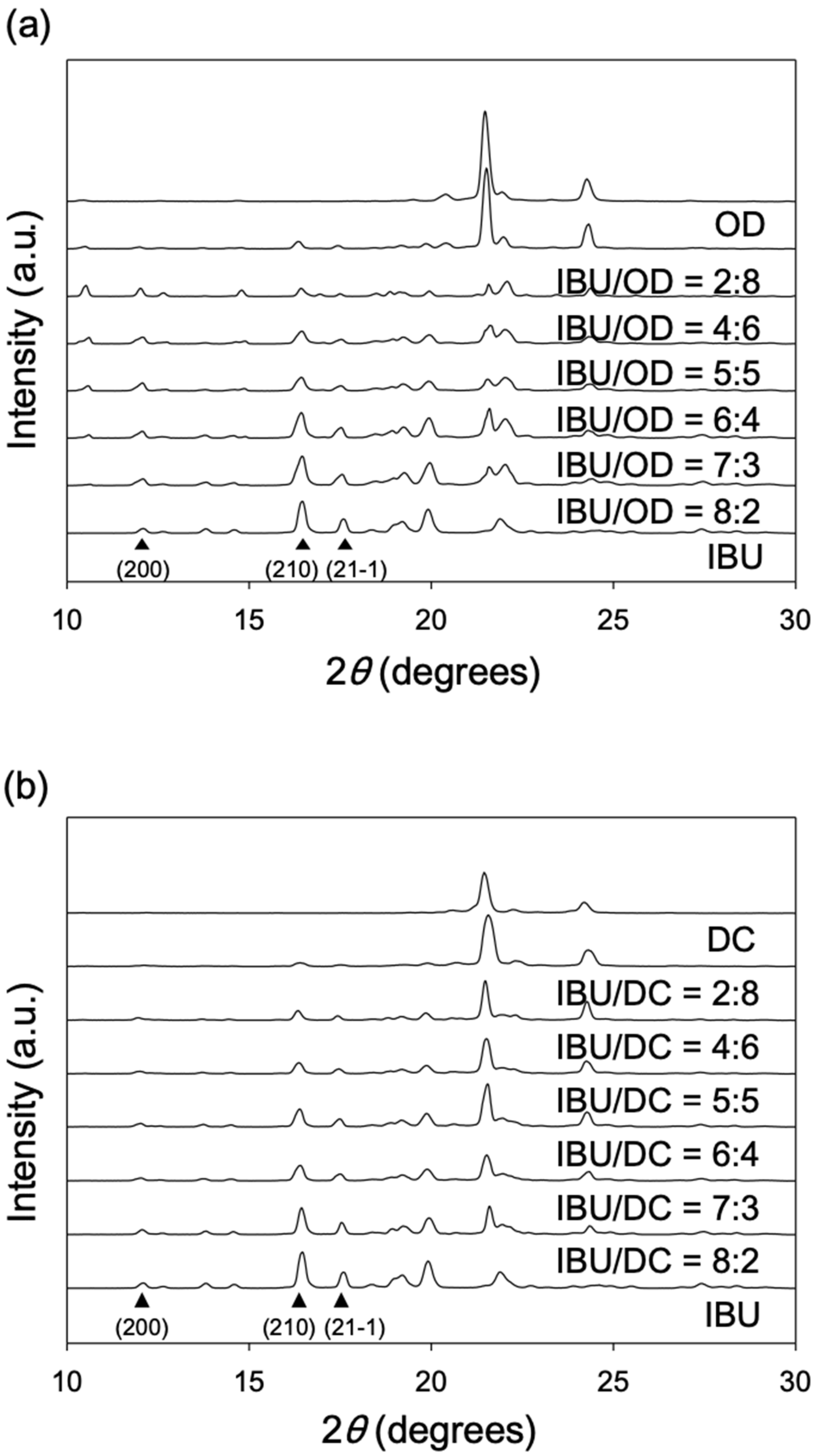

Figure S9. XRD patterns of (a) IBU/OD and (b) IBU/DC mixtures.

pharmaceutics

MDPI

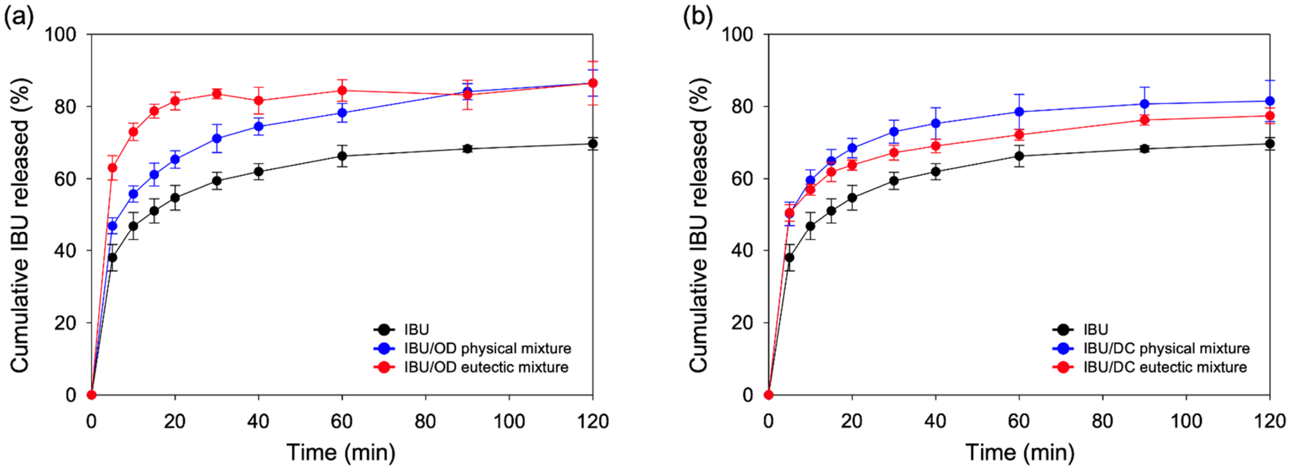

Figure S10. Dissolution profiles (FeSSIF, n = 3) of (a) IBU/OD eutectic mixture (5:5) and (b) IBU/DC eutectic mixture (6:4) in comparison of physical mixtures and pure IBU.
